# Edge states of Floquet–Dirac semimetal in a laser-driven semiconductor quantum-well

**DOI:** 10.1038/s41598-021-82230-3

**Published:** 2021-02-03

**Authors:** Boyuan Zhang, Nobuya Maeshima, Ken-ichi Hino

**Affiliations:** 1 Doctoral Program in Materials Science, Graduate School of Pure and Applied Sciences, University of Tsukuba, Tsukuba, Ibaraki 305-8573 Japan; 2 Center for Computational Sciences, University of Tsukuba, Tsukuba, 305-8577 Japan; 3 Division of Materials Science, Faculty of Pure and Applied Sciences, University of Tsukuba, Tsukuba, 305-8573 Japan

**Keywords:** Topological insulators, Two-dimensional materials, Topological insulators, Two-dimensional materials

## Abstract

Band crossings observed in a wide range of condensed matter systems are recognized as a key to understand low-energy fermionic excitations that behave as massless Dirac particles. Despite rapid progress in this field, the exploration of non-equilibrium topological states remains scarce and it has potential ability of providing a new platform to create unexpected massless Dirac states. Here we show that in a semiconductor quantum-well driven by a cw-laser with linear polarization, the optical Stark effect conducts bulk-band crossing, and the resulting Floquet-Dirac semimetallic phase supports an unconventional edge state in the projected one-dimensional Brillouin zone under a boundary condition that an electron is confined in the direction perpendicular to that of the laser polarization. Further, we reveal that this edge state mediates a transition between topological and non-topological edge states that is caused by tuning the laser intensity. We also show that the properties of the edge states are strikingly changed under a different boundary condition. It is found that such difference originates from that nearly fourfold-degenerate points exist in a certain intermediate region of the bulk Brillouin zone between high-symmetry points.

The theoretical prediction and the subsequent discovery of topological insulators^1,2^ have led to explosive expansion of the studies of topological perspectives of condensed matter^3,4^ and photonic crystals^5^, where a sharp distinction between topologically trivial and non-trivial phases with energy gaps is made by the presence of a gapless Dirac dispersion. The viewpoint of the gapless state has been developed to connect to the studies of topological semimetals akin to graphene^6–8^, termed Dirac, Weyl, and line-node semimetals^9,10^. Emergence of topological gapless phases is derived from symmetries inherent in the physical system of concern, namely, the time-reversal (T-)symmetry, the spatial-inversion (I-)symmetry, small groups supported by space groups, and so on^9–18^. As regards a Dirac semimetal (DSM), this is realized by an accidental band crossing due to fine-tuning of material parameters^11,12^, the symmetry-enforced mechanism^13,14^, and the band inversion mechanism^15–17^. Further, there exist edge modes known as double Fermi arcs at the surface of the DSM formed by the band inversion mechanism^17,19–21^. Recently, a growing attention has been paid to two-dimensional (2D) DSMs from the perspective of in-depth theories and applications to novel nano scale devices^14,18,22–24^.

While these intriguing topological semimetals are fabricated in equilibrium, there is still concealed attainability of creating and manipulating gapless Dirac dispersions in Floquet topological systems with spatiotemporal periodicity. Owing to this property, the existence of quasienergy bands are ensured by the Floquet theorem^25,26^. These systems are driven into non-equilibrium states by a temporally periodic external-field that has many degrees of freedom of controlling these states in terms of built-in parameters^27–36^. It is reported that a three-dimensional (3D) DSM, $\hbox{Na}_3\hbox{Bi}$, is changed to a Floquet-Weyl semimetal by irradiation of femtosecond laser pulses with a circularly polarized light^34^, and that band crossings at Dirac points are realized by forming a photonic Floquet topological insulator mimicking a graphene-like honeycomb lattice driven by a circularly polarized light^31^. It is remarked that the T-symmetry is broken/protected in a system under the application of a circularly/linearly polarized light-field.

In this study, first, we show that a gapless Dirac state emerges in a 2D-bulk band of a semiconductor quantum well driven by a cw-laser with a linear polarization, where the T-symmetry is protected, however, the I-symmetry is broken. Here, the optical Stark effect (OSE) accompanying quasienergy band splitting^37,38^ is introduced to cause an accidental band crossing at high-symmetry points in the 2D Brillouin zone (BZ). This effect is enhanced by a nearly resonant optical excitation from a valence (*p*-orbital) band to a conduction (*s*-orbital). Such an optically nonlinear excitation leads to strong hybridization between the different parity states with *s*- and *p*-orbitals over a wide range of the BZ due to the broken I-symmetry. Second, we show that such photoinduced hybridization brings the resulting DSM state to coincide with an unconventional edge state with a linear and nodeful dispersion in a projected one-dimensional (1D) BZ under a boundary condition that an electron is confined in the direction perpendicular to that of the applied electric field of laser. Further, when the laser intensity changes to make a gap open, this edge state is transformed smoothly into another edge state within this gap; which is either topologically trivial or non-trivial. It is also shown that the manifestation of these edge states is drastically changed under another boundary condition that an electron is confined in the direction parallel to the applied electric field. To deepen the understanding of the properties and boundary-condition dependence of the edge states, we introduce an interband polarization function that reflects the degree of parity hybridization in the bulk BZ. Finally, we point out that local anticrossings with quite small energy separation exist in a certain intermediate region of the 2D BZ between high-symmetry points, and show that these anticrossings leading to nearly fourfold degeneracy are crucial to understand the different properties of edge states, depending on the boundary conditions.

These edge states concerned here share features with other studies. As regards the OSE, a valley-selective OSE is demonstrated in monolayer transition metal dichalcogenides with application of a circularly polarized electric field^39^. As regards edge states of the Floquet DSM states, Tamm states^40–42^ appearing in the surfaces of several Dirac materials are theoretically examined^43^. Recently, growing interest has been captured in the interrelation of Tamm states with topological edge states in optical waveguide arrays^44–46^, 1D photonic crystals^47–49^, a graphene ring with the Aharonov-Bohm effect^50^, a honeycomb magnon insulator^51^, and a gold surface^52^.

## Results

### Modified Bernevig–Hughes–Zhang model with a laser-electron interaction

We begin by constructing the Hamiltonian of the present system of a semiconductor quantum well with a linearly polarized light field based on the paradigmatic Bernevig–Hughes–Zhang (BHZ) model^2^ composed of two bands with *s*- and *p*-orbitals in view of a spin degree of freedom. Hereafter, the band with *s*(*p*)-orbital is termed as *s*(*p*)-band just for the sake of simplicity, and the atomic units (a.u.) are used throughout unless otherwise stated. The BHZ Hamiltonian concerned here is read as the $4 \times 4$-matrix:

1 $$\begin{aligned} {\mathcal {H}}_{{\rm BHZ}}({\varvec{k}}) =\epsilon ({\varvec{k}})I+\sum _{i=3}^5 d_i({\varvec{k}})\Gamma _i \end{aligned}$$

with ${\varvec{k}}=(k_x, k_y)$ as a 2D Bloch momentum defined in the *xy*-plane normal to the direction of crystal growth of quantum well, namely, the *z*-axis. Here *I* represents the $4\times 4$ unit matrix, and $\Gamma _j$’s represent the four-dimensional Dirac matrices for the Clifford algebra, defined by $\Gamma _1=\tau _x\otimes \sigma _x,\, \Gamma _2=\tau _x\otimes \sigma _y,\, \Gamma _3=\tau _x\otimes \sigma _z,\, \Gamma _4=\tau _z\otimes I_2$, and $\Gamma _5=\tau _y\otimes I_2$, where $I_2$ represents the $2\times 2$ unit matrix, $\tau _s$ and $\sigma _s$ with $s=x,\,y,\,z$ represent the Pauli matrices for orbital and spin degrees of freedom, respectively, and the anti-commutation relation, $\{\Gamma _i, \Gamma _j \}=2\delta _{ij}$, is ensured. Further, $\epsilon ({\varvec{k}})={1\over 2}(\epsilon _s+\epsilon _p)-(t_{ss}-t_{pp})(\cos {k_x a}+\cos {k_y a})$, and

2 $$\begin{aligned} \left\{ \begin{array}{l} d_3({\varvec{k}})=2t_{sp}\sin {k_ya}\\ d_4({\varvec{k}})={1\over 2}(\epsilon _s-\epsilon _p)-(t_{ss}+t_{pp})(\cos {k_x a}+\cos {k_y a})\\ d_5({\varvec{k}})=2t_{sp}\sin {k_xa} \end{array} \right. , \end{aligned}$$

where $\epsilon _b$ and $8t_{bb}$ represent the center and width of band *b*, respectively, and $t_{bb^\prime }$ represents a hopping matrix between *b* and $b^\prime (\not = b)$ orbitals with lattice constant *a*; after this, *a* is set equal to unity unless otherwise stated. Hereafter, a semiconductor quantum well of HgTe/CdTe is accounted as the object of material. It is understood that $t_{ss}=t_{pp}$ and $\epsilon _s=-\epsilon _p$. Thus, a Fermi energy is given by $E_F=(\epsilon _s+\epsilon _p)/2=0$, and the energy gap $E_g$ at the $\Gamma$-point of the quantum well equals $2(\epsilon _s-4t_{ss})$.

An interaction of electron with a laser field is introduced into ${\mathcal {H}}_{{\rm BHZ}}({\varvec{k}})$ by replacing ${\varvec{k}}$ by ${\varvec{K}}(t)={\varvec{k}}+{\varvec{A}}(t)$, followed by adding to ${\mathcal {H}}_{{\rm BHZ}}({\varvec{K}}(t))$ an interband dipole interaction given by $v(t)=\Omega (t)\Gamma _6$, where $\Gamma _6=\tau _x\otimes I_2$, and $\Omega (t)$ is a real function of time *t*, provided as $\Omega (t)={\varvec{F}}(t)\cdot {\varvec{X}}_{sp}$. Here an electric field of the cw-laser with a linear polarization in the *x*-direction is given by ${\varvec{F}}(t)=(F_x\cos {\omega t},0,0)$ with a constant amplitude $F_x$ and a frequency $\omega$, where this is related with a vector potential ${\varvec{A}}(t)$ as ${\varvec{F}}(t)=-\dot{{\varvec{A}}}(t)$, and ${\varvec{X}}_{sp}=(X_{sp},0,0)$ represents a matrix element of electric dipole transition between *s*- and *p*-orbitals, independent of ${\varvec{k}}$: ${\varvec{X}}_{sp}={\varvec{X}}_{ps}^*$. Thus, in place of ${\mathcal {H}}_{{\rm BHZ}}({\varvec{k}})$, the resulting expression ends with up

3 $$\begin{aligned} H({\varvec{k}},t)={\mathcal {H}}_{{\rm BHZ}}({\varvec{K}}(t))+v(t) \equiv \sum _{i=3}^6D_i({\varvec{k}},t)\Gamma _i, \end{aligned}$$

where $D_i({\varvec{k}},t)=d_i({\varvec{K}}(t))$ for $i\not =6$, and $D_6({\varvec{k}},t)=\Omega (t)$ (for more detail of derivation of it, consult Supplementary Note 1). Obviously, this ensures the temporal periodicity, $H({\varvec{k}},t+T)=H({\varvec{k}},t)$, with $T=2\pi /\omega$, and the system of concern follows the Floquet theorem.

### T- and pseudo-I-symmetries

It is evident that the T- and I-symmetries are conserved in ${\mathcal {H}}_{\rm BHZ}({\varvec{k}})$, that is, $\Theta ^{-1}\, {\mathcal {H}}_{\rm BHZ}(-{\varvec{k}}) \Theta ={\mathcal {H}}_{{\rm BHZ}}({\varvec{k}})$, and $\Pi ^{-1}\, {\mathcal {H}}_{{\rm BHZ}}(-{\varvec{k}}) \Pi ={\mathcal {H}}_{{\rm BHZ}}({\varvec{k}})$, where $\Theta$ and $\Pi$ represent the T- and I-operators, defined by $\Theta =-iI_2\otimes \sigma _y K$ and $\Pi =\tau _z\otimes I_2$, respectively, where *K* means an operation of taking complex conjugate. Accordingly, by fine-tuning $E_g$, it is likely that an accidental band crossing occurs at a high-symmetry point with fourfold degeneracy^11^.

On the other hand, as regards $H({\varvec{k}},t)$, while the T-symmetry is still respected, the I-symmetry is broken because $D_i(-{\varvec{k}},t) \not =-D_i({\varvec{k}},t)$ for $i=5,6$, and $D_4(-{\varvec{k}},t) \not =D_4({\varvec{k}},t)$. That is, $\Theta ^{-1} H(-{\varvec{k}},-t) \Theta =H({\varvec{k}}, t)$, whereas $\Pi ^{-1} H(-{\varvec{k}},t) \Pi \not =H({\varvec{k}},t)$. In fact, it is shown that in terms of an operator defined as ${\tilde{\Pi }}=\Pi {\mathcal {T}}_{1/2}$, the symmetry ${\tilde{\Pi }}^{-1} H(-{\varvec{k}},t+T/2) {\tilde{\Pi }}=H({\varvec{k}},t)$ is retrieved, where ${\mathcal {T}}_{1/2}$ represents the operation of putting *t* ahead by a half period *T*/2, namely, the replacement of $t \rightarrow t+T/2$. Hereafter ${\tilde{\Pi }}$ is termed as the pseudo-I operator reminiscent of the time-glide symmetry^53^.

### Floquet quasienergy bands

Owing to the Floquet theorem, a wavefunction of the time-dependent Schr$\ddot{\mathrm{o}}$dinger equation for $H({\varvec{k}},t)$ is expressed as $\Psi _{{\varvec{k}}\alpha }(t)e^{-iE_\alpha ({\varvec{k}})t}$ for Floquet state $\alpha$, and thus $\Psi _{{\varvec{k}}\alpha }(t)$ is ensured by the quasi-stationary equation

4 $$\begin{aligned} L({\varvec{k}},t)\Psi _{{\varvec{k}}\alpha }(t)=E_\alpha ({\varvec{k}})\Psi _{{\varvec{k}}\alpha }(t) \end{aligned}$$

under a temporally periodic condition $\Psi _{{\varvec{k}}\alpha }(t+T)=\Psi _{{\varvec{k}}\alpha }(t)$, where $L({\varvec{k}},t)=H({\varvec{k}},t)-iI\partial /\partial t$ and $E_\alpha ({\varvec{k}})$ is an eigenvalue termed as quasienergy of the 2D bulk band. It is noted that $\Theta ^{-1} L(-{\varvec{k}},-t)\Theta =L({\varvec{k}},t)$, and ${\tilde{\Pi }}^{-1} L(-{\varvec{k}},t+T/2){\tilde{\Pi }}=L({\varvec{k}},t)$. The state $\alpha$ is denoted as a combination of $\beta (n)$, where $\beta$ is assigned to either *s*- or *p*-band that dominates over this hybridized state, and *n* represents an additional quantum number due to the temporal periodicity that means the number of dressing photons. Owing to the pseudo-I-symmetry, $E_{\alpha }({\varvec{k}})$ equals $E_{\alpha }(-{\varvec{k}})$, where the associated eigenstate of the former is $\Psi _{{\varvec{k}}\alpha }(t)$, while that of the latter is ${\tilde{\Pi }}\Psi _{{\varvec{k}}\alpha }(t)=\Psi _{-{\varvec{k}}\alpha }(t+T/2)$. It is remarked that a parity is still a good quantum number at a high-symmetry point ${\varvec{k}}={\varvec{k}}^j\,\,(j=\Gamma ,X_1,X_2,M)$, that is, $\Pi ^{-1} L({\varvec{k}}^j,t)\Pi =L({\varvec{k}}^j,t)$, where four *X*-points in the 2D-BZ are not equivalent because of the application of the laser field in the *x*-direction, and these are distinguished by representing as $X_1$ and $X_2$.

$E_{\alpha }({\varvec{k}})$’s are obtained by numerically solving Eq. (4) in the frequency $(\omega )$ domain, where the Floquet matrix is recast into ${\tilde{L}}_{nn^\prime }({\varvec{k}},\omega )=( n|L({\varvec{k}},t)|n^\prime )$ with respect to *n* and $n^\prime$ photon states; it is understood that $(n|\cdots |n^\prime )={1\over T}\int _{0}^T \,dt e^{-i(n-n^\prime )\omega t} \cdots$. The matrix element of it is read as

5 $$\begin{aligned} {\tilde{L}}_{nn^\prime }({\varvec{k}},\omega )=n\omega \delta _{nn^\prime }I +\sum _{i=3}^6 {\tilde{D}}_{i,nn^\prime }({\varvec{k}},\omega )\Gamma _i, \end{aligned}$$

where ${\tilde{D}}_{i,nn^\prime }({\varvec{k}},\omega )=( n|D_i({\varvec{k}},t)|n^\prime )$, and an explicit expression of it is given in Supplementary Note 2. A quasienergy band, ${\mathcal {E}}_{\alpha }(k_x)/{\mathcal {E}}_{\alpha }(k_y)$, which is the projection of $E_{\alpha }({\varvec{k}})$ onto the $k_x/k_y$-direction, is obtained by solving the equation provided by representing Eq. (4) in the lattice representation just in the *y*/*x*-direction where the motion of electron is confined. Thus, there are two types of vanishing boundary conditions that the electron is confined in the direction either perpendicular or parallel to the direction of ${\varvec{F}}(t)$. Hereafter, the former type is termed the boundary condition A, the latter is the boundary condition B; the allocation of both types is schematically shown in Supplementary Figure 1.

### Quasienergy-band inversion and crossing due to OSE

**Figure 1:**
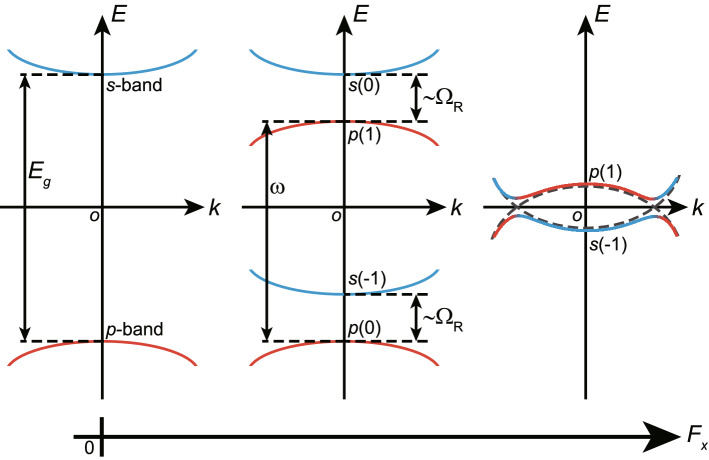
Scheme of the nearly resonant optical-excitation followed by the OSE. (Left) The original energy allocation of the *p*-band (red solid line) and the *s*-band (blue solid line) with energy gap $E_g$. (Center) With the application of cw-laser with frequency $\omega$ and constant electric field $F_x$, the OSE causes quasienergy-splitting of the order of the Rabi frequency $\Omega _R$ between a pair of photodressed bands, $s(n-1)$ and *p*(*n*), with $n=0,1$. (Right) With the further increase in $F_x$, a pair of bands of *p*(1) and $s(-1)$ undergoes inversion with anticrossing. Band crossing takes place at a certain $F_x$, as shown by a dashed line.

Here we show an overall change of quasienergy spectra with respect to $F_x$ due to the OSE, eventually leading to a band inversion. Figure 1 shows the scheme of the nearly resonant optical-excitation from the *p*-band to *s*-band with $\omega \lessapprox E_g$. Such a scheme of excitation almost maximizes the degree of the *sp* hybridization to induce sharp quasienergy-splitting of the order of $\Omega _R$ between two quasienergy bands of $s(n-1)$ and *p*(*n*) for $n=0,1$, where $\Omega _R$ represents the Rabi frequency given by $F_xX_{sp}$^37^. As $F_x$ increases, a pair of photodressed bands of *p*(1) and $s(-1)$ undergoes inversion to swerve with anticrossing.

**Figure 2:**
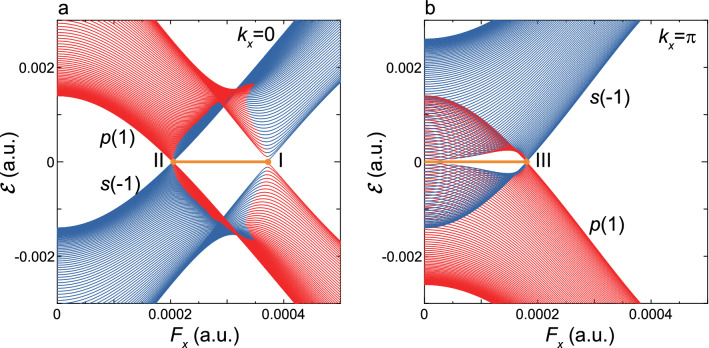
Band inversion and band crossing. (**a**) Shown are ${\mathcal {E}}_{p(1)}(k_x)$ and ${\mathcal {E}}_{s(-1)}(k_x)$ for $k_x=0$ as a function of $F_x$. The two quasienergy bands *p*(1) and $s(-1)$ (shown by red and blue lines, respectively) cross when $F_x$ is fine-tuned at the positions of I and II. Shown are the zero modes (Dirac nodes) by a yellow solid line. (**b**) The same as the (**a**) but for $k_x=\pi$. The two quasienergy bands cross when $F_x$ is fine-tuned at the position of III.

**Figure 3:**
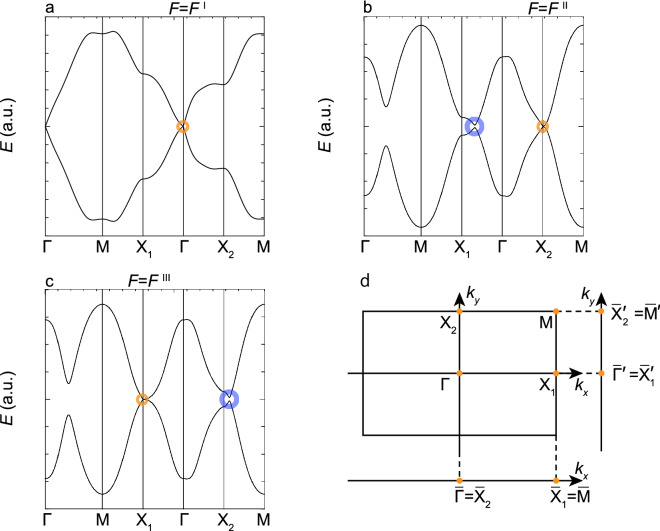
Quasienergy dispersion of 2D-bulk band $E({\varvec{k}})$. (**a**) Shown is $E({\varvec{k}})$ at $F_x^{{\rm I}}$, where $E_{p(1)}({\varvec{k}})$ and $E_{s(-1)}({\varvec{k}})$ are degenerate at the $\Gamma$ point (open yellow circle). (**b**) The same as the (**a**) but at $F_x^{{\rm II}}$ with the degeneracy at the $X_2$ point (open yellow circle) and the nearly degenerate valleys between the $\Gamma$ and $X_1$ points (open blue circle). (**c**) The same as the (**a**) but at $F_x^{{\rm III}}$ with the degeneracy at the $X_1$ point open yellow circle) and the nearly degenerate valleys between the $X_2$ and *M* points (open blue circle). (**d**) Shown are the high-symmetry points of $\Gamma , X_2, X_1$ and M in the 2D-BZ with their projection onto the $k_x$-axis denoted as ${\bar{\Gamma }}, {\bar{X}}_2, {\bar{X}}_1$ and ${\bar{M}}$, and onto the $k_y$-axis denoted as ${\bar{\Gamma }}^\prime , {\bar{X}}_2^\prime , {\bar{X}}_1^\prime$ and ${\bar{M}}^\prime$ respectively.

**Figure 4:**
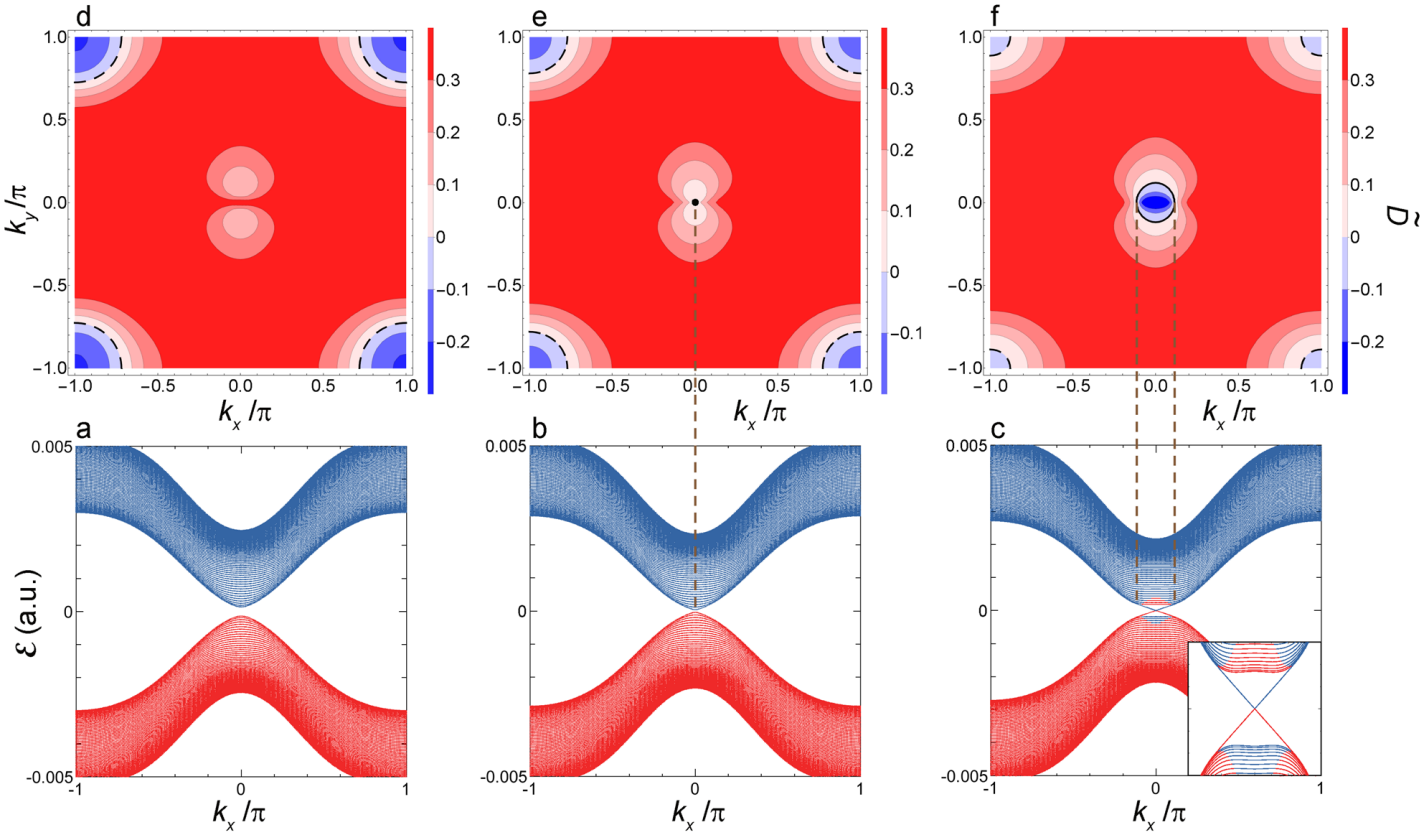
Quasienergy dispersion of $${\mathcal {E}}(k_x)$$ and interband polarization $$\tilde{{\mathcal {D}}}({\varvec{k}})$$ in the vicinity of $$F_x^{\Gamma }$$. (**a**) Shown are $${\mathcal {E}}_{p(1)}(k_x)$$ and $${\mathcal {E}}_{s(-1)}(k_x)$$ as functions of $$k_x$$ at $$F_x=3.82\times 10^{-4}\, (1.96\, \hbox{MV/cm})\, (F_x > F_x^{\Gamma })$$. The two quasienergy bands *p*(1) and $$s(-1)$$ are shown by red and blue lines, respectively. (**b**) The same as the (**a**) but at $$F_x^{\Gamma }=3.73\times 10^{-4}\, (1.92\, \hbox{MV/cm})$$. (**c**) The same as the (**a**) but at $$F_x=3.62\times 10^{-4}\, (1.86\, \hbox{MV/cm})\, (F_x^{X_2}< F_x < F_x^{\Gamma })$$. Inset: the expanded view of these two bands in the vicinity of the $${\bar{\Gamma }}$$-point. (**d**) Shown is a contour map $$\tilde{{\mathcal {D}}}({\varvec{k}})$$ in the $$(k_x,k_y)$$-plane at $$F_x $$ given by (**a**). Contours indicating the boundary of $$\tilde{{\mathcal {D}}}({\varvec{k}})=0$$ are shown by black dashed lines. (**e**) The same as the (**d**) but at $$F_x^{\Gamma }$$. Besides, a pinhole indicating $$\tilde{{\mathcal {D}}}({\varvec{k}})=0$$ at the $$\Gamma $$-point is shown by a black filled circle. The vertical dashed line shows the projection of $$\tilde{{\mathcal {D}}}({\varvec{k}})=0$$ (the pinhole) onto the $$k_x$$-axis shown in the (**b**). (**f**) The same as the (**d**) but at $$F_x$$ given by (**c**). Contours indicating the boundary of $$\tilde{{\mathcal {D}}}({\varvec{k}})=0$$ are shown by black solid and dashed lines. The vertical dashed lines show the projection of $$\tilde{{\mathcal {D}}}({\varvec{k}})=0$$ (the zero contour) onto the $$k_x$$-axis shown in the (**c**).

**Figure 5:**
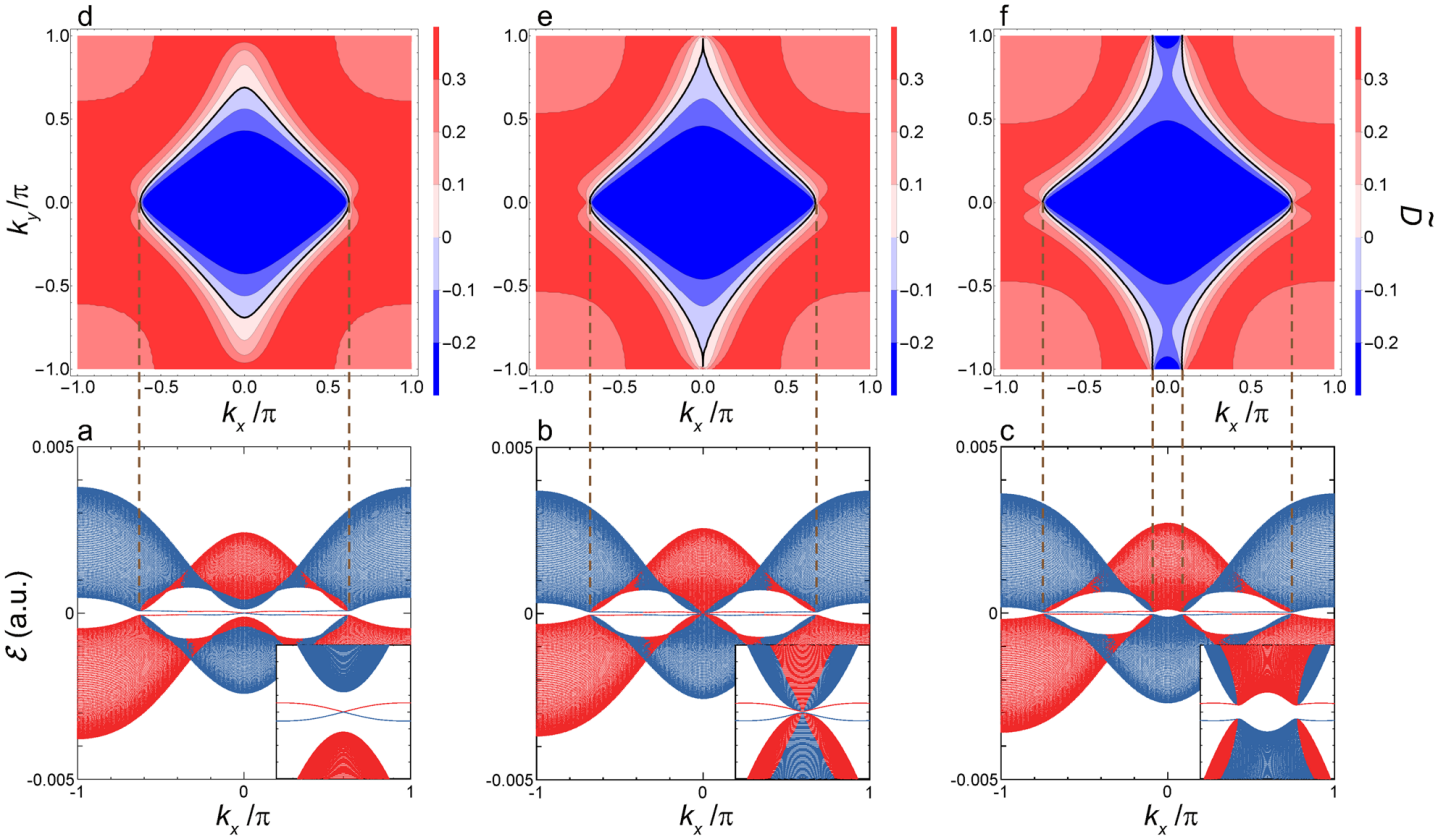
Quasienergy dispersion of ${\mathcal {E}}(k_x)$ and interband polarization $\tilde{{\mathcal {D}}}({\varvec{k}})$ in the vicinity of $F_x^{X_2}$. (**a**) Shown are ${\mathcal {E}}_{p(1)}(k_x)$ and ${\mathcal {E}}_{s(-1)}(k_x)$ as functions of $k_x$ at $F_x=2.14\times 10^{-4}\, (1.10\, \hbox{MV/cm})\,(F_x^{X_2}< F_x < F_x^\Gamma )$. The two quasienergy bands *p*(1) and $s(-1)$ are shown by red and blue lines, respectively. Inset: the expanded view of these two bands in the vicinity of the ${\bar{X}}_2$-point. (**b**) The same as the (**a**) but at $F_x^{X_2}=2.04\times 10^{-4}\, (1.05\, \hbox{MV/cm})$. (**c**) The same as the (**a**) but at $F_x =1.94\times 10^{-4}\, (997\, \hbox{kV/cm})\,(F_x^{X_1}< F_x < F_x^{X_2})$. (**d**) Shown is a contour map $\tilde{{\mathcal {D}}}({\varvec{k}})$ in the $(k_x,k_y)$-plane at $F_x$ given by (**a**). The vertical dashed lines show the projection of $\tilde{{\mathcal {D}}}({\varvec{k}})=0$ (the zero contour) onto the $k_x$-axis shown in the (**a**). (**e**) The same as the (**d**) but at $F_x^{X_2}$ and with the zero contour projected onto the $k_x$-axis shown in the (**b**). (**f**) The same as the (**d**) but at $F_x$ given by (**c**) and with the zero contour projected onto the $k_x$-axis shown in the (**c**).

**Figure 6:**
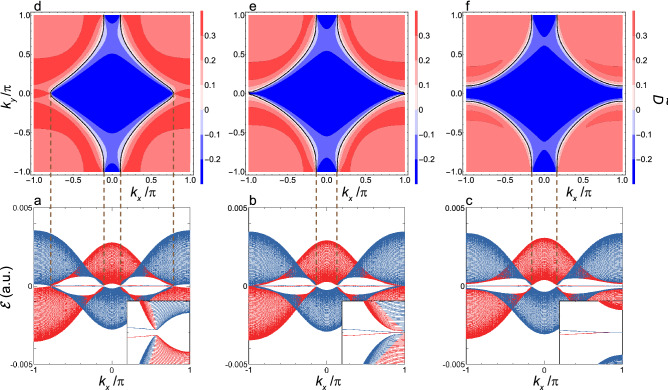
Quasienergy dispersion of ${\mathcal {E}}(k_x)$ and interband polarization $\tilde{{\mathcal {D}}}({\varvec{k}})$ in the vicinity of $F_x^{X_1}$. (**a**) Shown are ${\mathcal {E}}_{p(1)}(k_x)$ and ${\mathcal {E}}_{s(-1)}(k_x)$ as functions of $k_x$ at $F_x =1.89\times 10^{-4}\, (974\, \hbox{kV/cm})\,(F_x^{X_1}< F_x < F_x^{X_2})$. The two quasienergy bands *p*(1) and $s(-1)$ are shown by red and blue lines, respectively. Inset: the expanded view of these two bands in the vicinity of the ${\bar{X}}_1$-point. (**b**) The same as the (**a**) but at $F_x^{X_1} =1.80\times 10^{-4}\, (927\, \hbox{kV/cm})$. (**c**) The same as the (**a**) but at $F_x =1.69\times 10^{-4}\, (871\, \hbox{kV/cm})\,(F_x < F_x^{X_1})$. (**d**) Shown is a contour map $\tilde{{\mathcal {D}}}({\varvec{k}})$ in the $(k_x,k_y)$-plane at $F_x$ given by (**a**). The vertical dashed lines show the projection of $\tilde{{\mathcal {D}}}({\varvec{k}})=0$ (the zero contour) onto the $k_x$-axis shown in the (**a**). (**e**) The same as the (**d**) but at $F_x^{X_1}$ and with the zero contour projected onto the $k_x$-axis shown in the (**b**). (**f**) The same as the (**d**) but at $F_x$ given by (**c**) and with the zero contour projected onto the $k_x$-axis shown in the (**c**).

**Figure 7:**
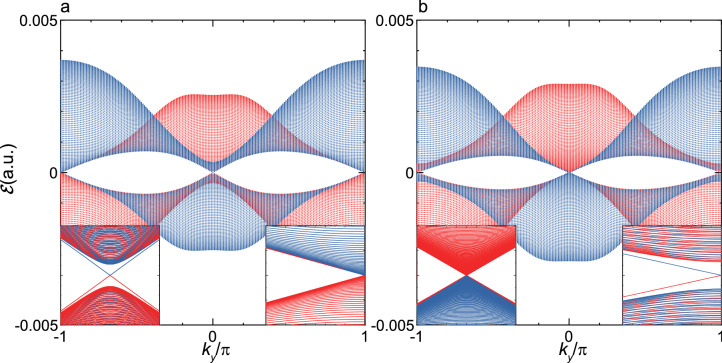
Quasienergy dispersion of ${\mathcal {E}}(k_y)$ at $F_x^{X_2}$ and $F_x^{X_1}$. (**a**) Shown are ${\mathcal {E}}_{p(1)}(k_y)$ and ${\mathcal {E}}_{s(-1)}(k_y)$ as functions of $k_y$ at $F_x^{X_2}=2.04\times 10^{-4}\, (1.05\, \hbox{MV/cm})$. The two quasienergy bands *p*(1) and $s(-1)$ are shown by red and blue lines, respectively. Insets: the expanded view of these two bands in the vicinity of the ${\bar{\Gamma }}^\prime ({\bar{X}}_1^\prime )$-point (left) and the ${\bar{X}}_2^\prime$-point (right). (**b**) The same as the (**a**) but at $F_x^{X_1}=1.80\times 10^{-4}\, (927\, \hbox{kV/cm})$.

Figure 2a, b show the calculated results of ${\mathcal {E}}_{p(1)}(k_x)$ and ${\mathcal {E}}_{s(-1)}(k_x)$ under the boundary condition A as a function of $F_x$ for $k_x=0$ and $\pi$, respectively. It is noted that these bands cross at the abscissa $({\mathcal {E}}(k_x)=E_F=0)$ without anticrossings at $F_x$’s indicated by I, II, and III; these positions are mentioned as $F_x^{{\rm I}}$, $F_x^{{\rm II}}$, and $F_x^{{\rm III}}$, respectively. The band inversions of *p*(1) and $s(-1)$ discerned in Fig. 2a, b accompany the emergence of zero-energy modes indicative of topological phase transitions, where the zero-energy modes are designated by the horizontal lines along the abscissa in $F_x^{{\rm II}}< F_x < F_x^{{\rm I}}$ and $F_x < F_x^{{\rm III}}$, respectively.

To examine the band crossings in detail, bulk bands $E({\varvec{k}})$ at $F_x^{{\rm I}}$, $F_x^{{\rm II}}$, and $F_x^{\rm III}$ are shown in Fig. 3a–c, where $E_{p(1)}({\varvec{k}})$ and $E_{s(-1)}({\varvec{k}})$ are degenerate at a single point of ${\varvec{k}}^j$ in the 2D-BZ with $j=\Gamma$, $X_2$, and $X_1$, respectively; these are indicated in Fig. 3d. Obviously, the crossing points seen in Fig. 2 are found identical with these high-symmetry points projected on the $k_x$-axis, which are denoted as ${\bar{\Gamma }}={\bar{X}}_2$ and ${\bar{X}}_1={\bar{M}}$. Actually, $E({\varvec{k}})$ is conical-shaped with linear-dispersion in the vicinity of ${\varvec{k}}^j$, and this is considered as a DSM state. It is understood that hereafter, $F_x^I, F_x^{II}$, and $F_x^{III}$ are represented as $F_x^\Gamma , F_x^{X_2}$, and $F_x^{X_1}$, respectively. These crossing points are also obtained by inspecting ${\mathcal {E}}_{p(1)}(k_y)$ and ${\mathcal {E}}_{s(-1)}(k_y)$ under the boundary condition B. The high-symmetry points projected on the $k_y$-axis, which are denoted as ${\bar{\Gamma }}^\prime ={\bar{X}}_1^\prime$ and ${\bar{X}}_2^\prime =\bar{M^\prime }$, are also depicted in Fig. 3d.

### Fourfold accidental degeneracy

Here we consider the origin of such band crossings. Because of the conservation of both T- and pseudo-I-symmetries, it is still probable that the band crossing between *p*(*n*) and $s(n^\prime )$ occurs at a high-symmetry point. In fact, to that end, an additional condition is required that the difference of photon numbers $\Delta n\equiv n-n^\prime$ is an even number. Contrariwise, when $\Delta n$ is odd, the resulting pair of bands are gapped out; especially, the two bands *p*(1) and *s*(0) never cross. Similarly to this case of the $\sigma _z$-conserving interactions, the above results still hold in the case of the $\sigma _z$-non-conserving interactions. All of the above conditions of band crossings are proved rigorously (see Supplementary Note 3).

### DSM states and edge states

First, we examine the 1D-band, ${\mathcal {E}}(k_x)$, and concomitant edge states obtained under the boundary condition A. These edge states are either topologically trivial or non-trivial; hereafter, it is understood that the term of the Tamm state^43^ is used exclusively to mean a trivial state bound on an edge to distinguish it from a non-trivial one. Figures 4a–c, 5a–c, and 6a–c show the spectra of ${\mathcal {E}}(k_x)$ in the decreasing order of $F_x$. It is seen that all the DSM states delimits the boundary of a topological phase transition (see Figs. 4b,  5b,  6b). It should be noted that the DSM states observed at $F_x^{X_2}$ and $F_x^{X_1}$ coincide with edge states with linear and nodeful dispersions (see Figs. 5b, 6b, respectively), differing from that observed at $F_x^{\Gamma }$ (see Fig. 4b). Such edge states are termed the Dirac–Tamm state hereafter just for the sake of convenience of making a distinction from other Tamm states. As regards the Dirac–Tamm state at $F_x^{X_2}$, with the slight increase of $F_x$ to make a gap open, this is transformed into an unequivocally topological edge state with its band structure kept almost as it stands (see Fig. 5a), while with the change of $F_x$ in the opposite direction, this becomes nodeless with two flat dispersions (see Fig. 5c). As regards the Dirac–Tamm state at $F_x^{X_1}$, with the slight increase of $F_x$, this is transformed into a nodeless edge state (see Fig. 6a), while with the slight decrease of $F_x$, this becomes unequivocally topologically trivial (see Fig. 6c).

Next, we examine the 1D-band, ${\mathcal {E}}(k_y)$, and concomitant edge states obtained under the boundary condition B. Figure 7 shows the two representative quasienergy bands at $F_x^{X_2}$ and $F_x^{X_1}$. Differing from the quasienergy bands shown in Fig. 5b/6b, a Dirac–Tamm state is faint and undiscernible at the ${\bar{X}}_2^\prime /{\bar{X}}_1^\prime$ point, though a linear and nodeful dispersion is still discernible around the ${\bar{X}}_1^\prime /{\bar{X}}_2^\prime$. According to these results, it is evident that the specification of the imposed boundary condition is crucial for the discussion of the edge states. Discussion of the origin of such difference will be deepened below.

The topological nature of these edge states is evaluated in terms of the Chern number of the lower band, denoted as $\alpha _L$, where $E_{\alpha _L}({\varvec{k}})\le E_F=0$; this number is independent of the boundary conditions. It is confirmed that the non-zero values of $C_{\alpha _L}=1$ are obtained in $F_x^{X_2}< F_x < F_x^{\Gamma }$ and $F_x < F_x^{X_1}$, otherwise this vanishes. Thus, we verify that the edge state observed in $F_x^{X_1}< F_x < F_x^{X_2}$ is a Tamm state (see Figs. 5c,  6a). Further, the Dirac–Tamm states at $F_x^{X_1}$ and $F_x^{X_2}$ are also considered Tamm states, since their respective net Chern numbers are zero^10^.

### Interband polarization

To understand the manifestation of the edge states seen in Figs. 4, 5, 6 and 7 and the boundary-condition dependence, a macroscopic polarization of the present system, that is, an induced dipole moment, is examined. This is given by

6 $$\begin{aligned} D_{{\varvec{k}}\alpha _L}(t) =\langle \Psi _{{\varvec{k}}\alpha _L}(t)|x|\Psi _{{\varvec{k}}\alpha _L}(t)\rangle =\sum _{bb^\prime (b\not = b^\prime )}[P_{{\varvec{k}}\alpha _L}(t)]_{bb^\prime }X_{b^\prime b} \end{aligned}$$

for state $\alpha _L$, where *x* is the *x*-component of position vector of electron. Here, $P_{{\varvec{k}}\alpha _L}(t)$ represents the associated microscopic interband polarization corresponding to an off-diagonal element of a reduced density matrix, and $[P_{{\varvec{k}}\alpha _L}(t)]_{sp}=[P_{{\varvec{k}}\alpha _L}(t)]_{ps}$ because of $X_{sp}=X_{ps}$^54^. The interband polarization in the $\omega$-domain is introduced as: ${\tilde{P}}^{(N)}_{{\varvec{k}}\alpha _L}(\omega ) =(0|D_{{\varvec{k}}\alpha _L}(t)|N)/X_{sp}$ with ${\tilde{P}}^{(-N)}_{{\varvec{k}}\alpha _L}(\omega )= [{\tilde{P}}^{(N)}_{{\varvec{k}}\alpha _L}(\omega )]^*$. Below, we examine $\tilde{{\mathcal {D}}}({\varvec{k}}) \equiv {\hbox{Re}}[{\tilde{P}}^{(1)}_{{\varvec{k}}\alpha _L}(\omega )]$ as a function of ${\varvec{k}}$ in the 2D-BZ; neither ${\tilde{P}}^{(N\not = \pm 1)}_{{\varvec{k}}\alpha _L}(\omega )$ nor ${\hbox{Im}}[{\tilde{P}}^{(\pm 1)}_{{\varvec{k}}\alpha _L}(\omega )]$ show significant variance in the BZ with the change in $F_x$. It is stated that $\tilde{{\mathcal {D}}}({\varvec{k}})$ precisely reflects the degree of parity hybridization in the 2D-BZ that results from the I-symmetry breaking.

The calculated results of $\tilde{{\mathcal {D}}}({\varvec{k}})$ are shown in Figs. 4d–f, 5d–f, and 6d–f along with ${\mathcal {E}}(k_x)$ in the vicinity of $F_x^{\Gamma }, F_x^{X_2}$, and $F_x^{X_1}$, respectively, where a black solid line shows a contour indicating the boundary of $\tilde{{\mathcal {D}}}({\varvec{k}})=0$, which is hereafter termed as the zero contour. It is readily seen that the zero-contour projected onto the $k_x$-axis coincides with the segment of the 1D-BZ at which an edge state manifests itself irrespective of being topological or not. To be more specific, the edge state is discerned where a vertical line that is parallel to the $k_y$-axis at a certain $k_x$ crosses the zero contour twice. For instance, as seen in Fig. 6f, the vertical line crosses this contour twice except around the ${\bar{\Gamma }}$-point, and the edge state emerges in the corresponding range of $k_x$. It is remarked that another contour indicating $\tilde{{\mathcal {D}}}({\varvec{k}})=0$ is discerned around the *M*-points in Fig. 4d–f, which is shown by a black dashed line; this causes no edge state and is attributed to an anticrossing between bands of $s(-1)$ and *p*(2) where the difference of the respective photon numbers is an odd number.

The above relation of the zero contour with the formation of edge state is straightforward applied to the case of the boundary condition B. By projecting $\tilde{{\mathcal {D}}}({\varvec{k}})$’s shown in Figs. 4, 5 and 6 onto the $k_y$-direction, the existence of edge states and their forms of manifestation are examined. According to this, it is speculated that in the regions of $F_x >F_x^{X_2}$ and $F_x <F_x^{X_1}$, edge states with the similar patterns to those in the case of the boundary condition A emerge. In the rest of the regions, edge states exist with the shape of $\infty$ having nodes and antinodes in the whole 1D-BZ. The various forms of these edge states are schematically depicted in Supplementary Figure 2. By comparing the 1D-bands shown in Fig. 7a, b with the above-speculated results, it is found that most parts of the $\infty$-shaped edge states are merged into the bulk continuum, and the Dirac–Tamm states are not discernible around the ${\bar{X}}_2^\prime$ and ${\bar{X}}_1^\prime$ points, respectively; though the linear and nodeful dispersions remain just around the ${\bar{X}}_1^\prime$ and ${\bar{X}}_2^\prime$ points, respectively. Therefore, it is stated that the Dirac–Tamm states exist in the case of the boundary condition A, whereas not in the case of the boundary condition B.

### Nearly fourfold degeneracy and boundary-condition dependence

Here we examine the origin of the above-mentioned boundary-condition dependence, based on the two anticrossings located in between the $X_1$ and $\Gamma$ points and the $X_2$ and *M* points seen in Fig. 3b and c, respectively. The I-symmetry breaking causes band anisotropy of $E({\varvec{k}})$, namely, the dependence of band width on the direction of ${\varvec{k}}$, to form the anticrossing near the $X_{1(2)}$-point when a gap closes at the $X_{2(1)}$-point. Even if the gap opens, the anticrossing is sustained in a certain range of $F_x$ with moving in the 2D-BZ, differing from the accidental fourfold degeneracies at the high-symmetry points; these are lifted by slight changes of $F_x$.

Such a property is seen in Figs. 5 and 6, as follows. The anticrossing along the $X_1-\Gamma$ line is found in ${\mathcal {E}}(k_x)$’s shown in Figs. 5a–c and 6a, and merges into the crossing at the ${\bar{X}}_1$ point, as shown in Fig. 6b. The anticrossing along the $X_2-M$ line is found in ${\mathcal {E}}(k_x)$’s shown in Figs. 5c and 6a–c, and merges into the crossing at the ${\bar{X}}_2$ point, as shown in Fig. 5b. Thus, these anticrossings are stable against the change of $F_x$ and look nearly fourfold degenerate because of quite small energy separation of the order of 1meV. Hereafter, for the sake of convenience, very local regions of ${\varvec{k}}$ over which the anticrossings extend along the $X_1-\Gamma$ and $X_2-M$ lines are termed $V_1$ and $V_2$ points, respectively; further the terms of ${\bar{V}}_1$ and ${\bar{V}}_2$ points are used as the projection onto the $k_x$-direction, respectively.

The singular property around the $V_1$ and $V_2$ points is confirmed by seeing the variance of $\tilde{{\mathcal {D}}}(k_x,0)$ and $\tilde{{\mathcal {D}}}(k_x,\pm \pi )$ with respect to $k_x$. For instance, in Fig. 5f, these functions traverse the zero contours with steep changes at the $V_1$ and $V_2$ points, respectively (see Supplementary Figure 3). Such behavior is attributed to an adiabatic interchange of the constituent of wavefunction $\Psi _{{\varvec{k}}\alpha _L}(t)$ between *p*(1) and $s(-1)$ at these points. This makes $\Psi _{{\varvec{k}}\alpha _L}(t)$ almost discontinuous, leading to an abrupt change of parity with the traverse of $k_x$ at these points. In other words, diabolic-like points are formed at the $V_1$ and $V_2$ points as if monopoles of Berry curvature existed^55^.

The existence of the nearly fourfold degeneracies causes more involved edge-state structure within the gap in ${\mathcal {E}}_\alpha (k_x)$ than that in ${\mathcal {E}}_\alpha (k_y)$. By connecting the points of ${\bar{V}}_1$ and ${\bar{V}}_2$ in different manners, all of the topological edge states, the Dirac–Tamm states, and Tamm states seen in Figs. 5a–c and 6a–c are formed in the close vicinity of ${\mathcal {E}}_\alpha (k_x)=E_F$, whether there is a Dirac node or not in an edge state. Besides their topological natures depending on the change of $F_x$, all these edge states are considered to have the same properties pertinent to the degree of localization of confined electron owing to the same manner of formation; though the Dirac–Tamm states become delocalized just around a local $k_x$-region where bands cross, due to couplings with continuum of Floquet DSM phases (see Supplementary Note 4). On the other hand, just topological edge states manifest themselves in ${\mathcal {E}}_\alpha (k_y)$ without the effect of the nearly fourfold degeneracies at the points of $V_1$ and $V_2$ (see Fig. 7 and Supplementary Figure 2). Therefore, it is concluded that the existence of the nearly fourfold degeneracies is a key effect which governs the manifestation of the Dirac–Tamm states and the Tamm states under the boundary condition A.

## Discussion

This work shows that the nearly resonant laser-excitation combined with the OSE gives rise to the fourfold accidental degeneracies at the high-symmetry points, and the resulting Floquet DSM states host unconventional Dirac–Tamm states that are transformable into either topological edge states or Tamm states with the change of $F_x$ just under the boundary condition A, differing from the results under the boundary condition B. A stress is put on the existence of the nearly fourfold degeneracies at the $V_1$ and $V_2$ points that arise from the I-symmetry breaking, because these remain stable against the change of $F_x$ in a certain region of it and fulfills the key role of understanding the different boundary-condition dependence of the edge states. Such boundary-condition dependence of the present system is reminiscent of graphene with the zigzag and armchair boundary conditions^6–8^; actually, the Tamm states have almost flat energy dispersion connecting the two points of $V_1$ and $V_2$, while graphene has a zigzag edge state with a flat dispersion between the *K* and $K^\prime$ points.

In addition, this study is also related with rapidly noticed studies on the interrelation between a Tamm state and a topological edge state, because the state-of-the-art techniques of fabrication of optical waveguide arrays and photonic crystals have made it possible to create both edge states by mimicking the one-dimensional Su-Schriefer-Hegger model^44–46,49,56^ and more complicated systems^47,48,50^. In this study, both of the edge states are transformed in a continuous manner as a function of the single parameter $F_x$ without changing the composition and structure of the system, which draws a sharp distinction from these existing studies.

Finally, we make comments on the possibility of observing the present findings. Here, the variance of $F_x$ is around the order of 1MV/cm, leading to high-density electron excitation with dephasing and population relaxation times of the order of a few hundred fs. Thus, ultrashort pulse irradiation with $\omega \approx 300$ meV ($T\approx 14$ fs) and temporal width of the order of 100 fs is required for realizing band inversion between $E_{s(-1)}({\varvec{k}})$ and $E_{p(1)}({\varvec{k}})$ to form various types of the edge states. It would be possible to confirm the manifestation of these states by virtue of the optoelectronic technique of measuring quasimetallic photoconductivity produced by pulse irradiation^57^, which has been utilized for the time-resolved measurement of light-induced Hall effect in graphene^58,59^. In addition, it is remarked that due to the many-body Coulomb interaction resulting from intense photoexcitation of electrons, the Floquet bands and the values of $F_x$ at which the band inversion and crossing occur are somewhat modified by the renormalization of carrier energy and Rabi energy^54^.

## Methods

Numerical calculations for a wavefunction $\Psi _{{\varvec{k}}\alpha }(t)$ of Floquet state $\alpha$ and the associated quasienergy $E_{\alpha }({\varvec{k}})$ are implemented by relying on the Fourier-Floquet expansion of Eq. (4), followed by diagonalizing the Floquet matrix ${\tilde{L}}_{nn^\prime }({\varvec{k}},\omega )$. The explicit expressions of matrix elements of it are given in Supplementary Note 2. The maximum number of photons $(N_p)$ incorporated in this calculation is three, namely, $n, n^\prime =-N_p \sim N_p$, and the numerical convergence is checked by using a greater value of $N_p$. The following material parameters in the units of a.u. are employed for actual calculations^60^: $\epsilon _s=-\epsilon _p=0.01, t_{ss}=t_{pp}=0.001, t_{sp}=0.002, a=12.21$, and $X_{sp}=34.63$. $\omega$ and $E_g$ are set to be 0.0114 and 0.012, respectively. Further, the Chern number of a lower band $\alpha _L$ is evaluated by calculating

7 $$\begin{aligned} C_{\alpha _L}={1\over 2\pi }\oint d{\varvec{k}}\cdot {\varvec{a}}_{\alpha _L}({\varvec{k}}), \end{aligned}$$

where the Berry connection is defined by ${\varvec{a}}_{\alpha _L}({\varvec{k}})=-{i\over T}\int ^T_0dt\, \langle \Psi _{{\varvec{k}}\alpha _L}(t)|\nabla _{{\varvec{k}}}\Psi _{{\varvec{k}}\alpha _L}(t)\rangle$.

## Supplementary information

Supplementary information.

## Data Availability

Data sharing not applicable to this article as no datasets were generated or analysed during the current study.

## Code availability

No use of custom code or mathematical algorithm that is deemed central to the conclusions of the current study.
