# Supplementary material for: Edge states of Floquet–Dirac semimetal in a laser-driven semiconductor quantum-well
Source: Sci Rep. 2021 Feb 3;11:2952. doi: 10.1038/s41598-021-82230-3 (PMC7859246; doi:10.1038/s41598-021-82230-3)
Supplement: Supplementary file 1 — Supplementary information. [file 41598_2021_82230_MOESM1_ESM.pdf]

Supplementary Information for Scientific Reports:

" Edge states of Floquet-Dirac semimetal in a laser-driven semiconductor quantum-well "

Boyuan Zhang, Nobuya Maeshima, and Ken-ichi Hino
University of Tsukuba, Japan

Contents

1 Supplementary Figure 1: Scheme of edges of the semiconductor quantum well 2

2 Supplementary Figure 2: Variance of interband polarization $\tilde{\mathcal{D}}(\boldsymbol{k})$ and boundary-condition dependent edge states 3

3 Supplementary Figure 3: Two dimensional sections of the plots of interband polarization $\tilde{\mathcal{D}}(\boldsymbol{k})$ with respect to k_x 4

4 Supplementary Note 1: Derivation of the modified Bernevig-Hughes-Zhang (BHZ) Hamiltonian 5

5 Supplementary Note 2: Fourier-Floquet matrix elements 7

6 Supplementary Note 3: Additional condition of quasienergy band crossing 8

6.1 A σ_z-conserving interaction 8

6.2 A σ_z-non-conserving interaction 9

6.3 Summary 10

7 Supplementary Note 4: The degree of localization of the edge states in the vicinity of $F_x^{X_2}$ 11

1 Supplementary Figure 1: Scheme of edges of the semiconductor quantum well

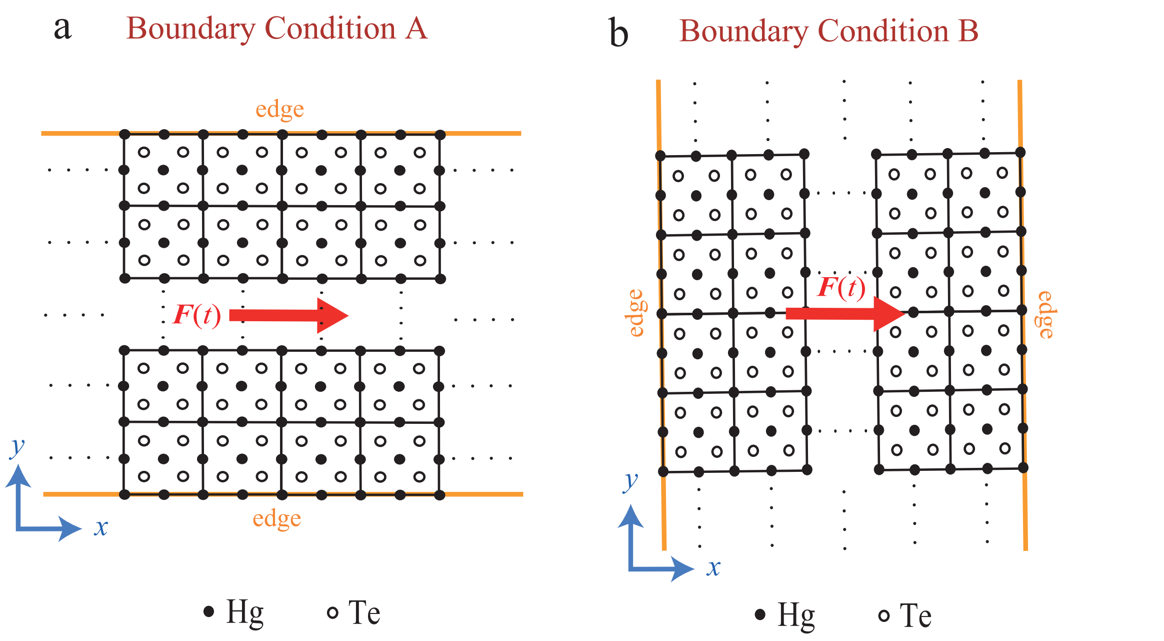

Figure 1: **Scheme of edges of the semiconductor quantum well.** **(a)** The boundary condition A is set along the edge in the x-direction, where an electron is confined in the y-direction perpendicular to the electric field of lase $\boldsymbol{F}(t)$ (the x-direction), and the system of concern, namely, the quantum wire, is spatially periodic in the x-direction. **(b)** The boundary condition B is set along the edge in the y-direction, where an electron is confined in the x-direction parallel to $\boldsymbol{F}(t)$, and the system of concern is spatially periodic in the y-direction. In these figures, an electron is confined in a well layer of the quantum well (HgTe) with zinc blende structure the unit cell of which is depicted.

2 Supplementary Figure 2: Variance of interband polarization $\tilde{\mathcal{D}}(\boldsymbol{k})$ and boundary-condition dependent edge states

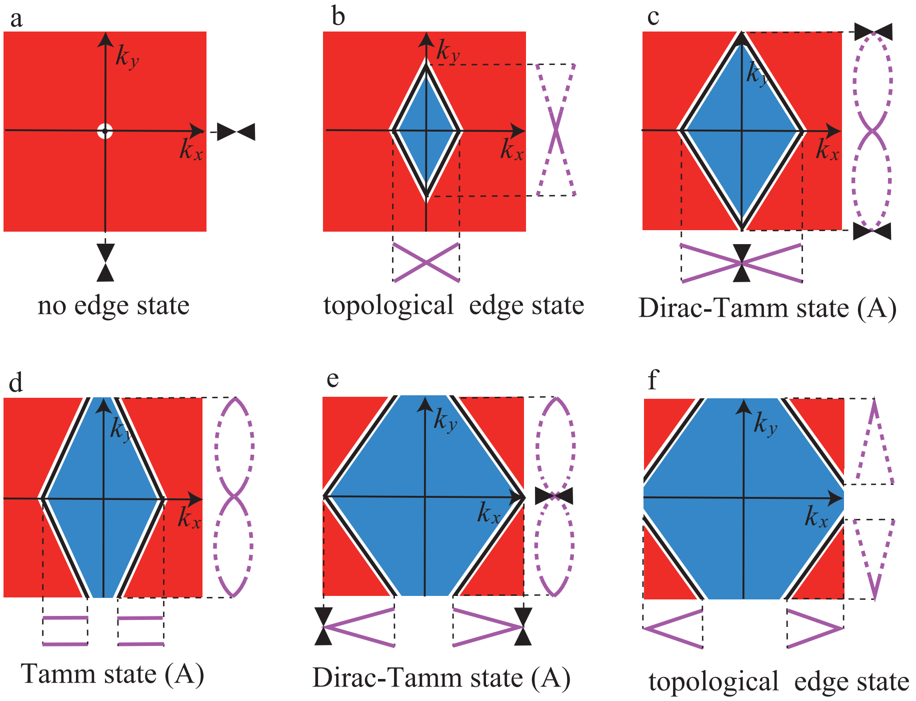

Figure 2: **Schematic view of interband polarization $\tilde{\mathcal{D}}(\boldsymbol{k})$ and edge states with the change of F_x.** Here, shown are schematic contour-maps of $\tilde{\mathcal{D}}(\boldsymbol{k})$ in the (k_x, k_y)-plane, where regions with $\tilde{\mathcal{D}}(\boldsymbol{k}) > 0$ and $\tilde{\mathcal{D}}(\boldsymbol{k}) < 0$ are indicated by red and blue colors, respectively, a transient region between the two is indicated by a white color, and the boundary of $\tilde{\mathcal{D}}(\boldsymbol{k}) = 0$ (the zero contour) is indicated by a black solid line. Further, edge states revealed in one-dimensional energy dispersions $\mathcal{E}(k_x)$ (under the boundary condition A) and $\mathcal{E}(k_y)$ (under the boundary condition B) are schematically shown by purple solid lines; parts of edges states embedded in a bulk band continuum are indicated by a purple dashed line. In addition, when a Dirac crossing is caused, the resulting Dirac cone projected onto the k_x and k_y axes is shown by intersected filled triangles. **(a)** $\tilde{\mathcal{D}}(\boldsymbol{k})$ is shown at F_x^{Γ}. Here edge states are absent. **(b)** $\tilde{\mathcal{D}}(\boldsymbol{k})$ is shown at F_x where $F_x^{X_2} < F_x < F_x^{\Gamma}$. The black dashed lines show the projection of the zero contour onto the k_x and k_y axes, and these delimit the edge states appearing in $\mathcal{E}(k_x)$ and $\mathcal{E}(k_y)$, respectively. Here topological edge states manifest themselves. **(c)** The same as the panel (b) but at $F_x^{X_2}$. Here Dirac-Tamm states manifest themselves just under the boundary condition A. **(d)** The same as the panel (b) but at F_x where $F_x^{X_1} < F_x < F_x^{X_2}$. Here Tamm states manifest themselves just under the boundary condition A. **(e)** The same as the panel (b) but at $F_x^{X_1}$. Here Dirac-Tamm states manifest themselves just under the boundary condition A. **(f)** The same as the panel (b) but at F_x where $F_x < F_x^{X_1}$. Here topological edge states manifest themselves.

3 Supplementary Figure 3: Two dimensional sections of the plots of interband polarization $\tilde{\mathcal{D}}(\boldsymbol{k})$ with respect to k_x

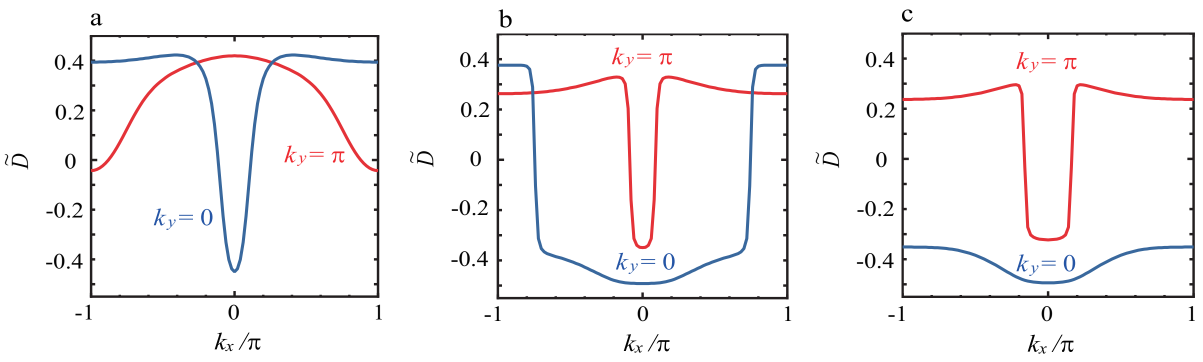

Figure 3: **Two dimensional sections of the plots of interband polarization $\tilde{\mathcal{D}}(\boldsymbol{k})$ with respect to k_x.** Interband polarization $\tilde{\mathcal{D}}(\boldsymbol{k})$ as a function of k_x with k_y fixed at 0 (blue) and π (red) at F_x equal to **(a)** 3.62×10^{-4} a.u. (1.86 MV/cm) corresponding to Fig. 4f in the text. **(b)** 1.94×10^{-4} a.u. (997 kV/cm) corresponding to Fig. 5f in the text. **(c)** 1.69×10^{-4} a.u. (871 kV/cm) corresponding to Fig. 6f in the text.

4 Supplementary Note 1: Derivation of the modified Bernevig-Hughes-Zhang (BHZ) Hamiltonian

We begin with the Schrödinger equation

$$\hat{H}(t)|\Psi\rangle = i\frac{\partial}{\partial t}|\Psi\rangle, \tag{1}$$

with the total Hamiltonian as $\hat{H}(t) = \hat{H}^0 + \hat{H}'(t)$ at time t, where $\hat{H}^0$ and $\hat{H}'(t)$ represent a material Hamiltonian and a Hamiltonian of a laser-electron electric-dipole interaction, respectively. These are given in terms of a Wannier state $|b\boldsymbol{R}\rangle$ with b and $\boldsymbol{R}$ a band index and a two-dimensional site vector of the system of concern (a semiconductor quantum well) as follows:

$$\hat{H}^0 = \sum_{b,\boldsymbol{R},b',\boldsymbol{R}'} |b\boldsymbol{R}\rangle H^0_{b\boldsymbol{R},b'\boldsymbol{R}'}\langle b'\boldsymbol{R}'|, \tag{2}$$

and

$$\hat{H}'(t) = \sum_{b,\boldsymbol{R},b',\boldsymbol{R}'} |b\boldsymbol{R}\rangle H'_{b\boldsymbol{R},b'\boldsymbol{R}'}(t)\langle b'\boldsymbol{R}'|. \tag{3}$$

in terms of the corresponding matrix elements of $H^0_{b\boldsymbol{R},b'\boldsymbol{R}'}$ and $H'_{b\boldsymbol{R},b'\boldsymbol{R}'}(t)$, respectively. Here, the matrix element $H'_{b\boldsymbol{R},b'\boldsymbol{R}'}(t)$ is given by

$$H'_{b\boldsymbol{R},b'\boldsymbol{R}'}(t) = \langle b\boldsymbol{R}|\boldsymbol{F}(t)\cdot\boldsymbol{r}|b'\boldsymbol{R}'\rangle = \boldsymbol{F}(t)\cdot\boldsymbol{R}\,\delta_{bb'}\delta_{\boldsymbol{R}\boldsymbol{R}'} + \boldsymbol{F}(t)\cdot\boldsymbol{X}_{bb'}\bar{\delta}_{bb'}\delta_{\boldsymbol{R}\boldsymbol{R}'}. \tag{4}$$

In the first equality, $\boldsymbol{F}(t)$ represents an electric field vector of an irradiated laser and $\boldsymbol{r}$ represents the position vector of electron. In the second equality, $\boldsymbol{X}$ is defined as the displacement of electron from the position of an ionic core of the quantum well, namely, $\boldsymbol{X} = \boldsymbol{r} - \boldsymbol{R}$, and an electric dipole matrix element at each $\boldsymbol{R}$ is approximately given by $\langle b\boldsymbol{R}|\boldsymbol{X}|b'\boldsymbol{R}'\rangle \approx \boldsymbol{X}_{bb'}\delta_{\boldsymbol{R}\boldsymbol{R}'}$ with $\boldsymbol{X}_{bb'} = \langle b|\boldsymbol{X}|b'\rangle$. To do this, the property has been used that a Wannier function $\langle\boldsymbol{r}|b\boldsymbol{R}\rangle$ is just a function of $\boldsymbol{X}$, and mostly localized inside a cell situated at $\boldsymbol{R}$. Thus, $\langle b\boldsymbol{R}|\boldsymbol{X}|b'\boldsymbol{R}'\rangle$ is considered almost independent of $\boldsymbol{R}$. Therefore, Eq. (1) is cast into

$$\sum_{b'\boldsymbol{R}'}\left\{H^0_{b\boldsymbol{R},b'\boldsymbol{R}'} + \boldsymbol{F}(t)\cdot(\boldsymbol{R}\delta_{bb'} + \boldsymbol{X}_{bb'}\bar{\delta}_{bb'})\delta_{\boldsymbol{R}\boldsymbol{R}'}\right\}\langle b'\boldsymbol{R}'|\Psi\rangle = i\frac{\partial}{\partial t}\langle b\boldsymbol{R}|\Psi\rangle. \tag{5}$$

Now, the Peierls phase transform defined by

$$|\Psi\rangle = e^{i\boldsymbol{A}(t)\cdot\boldsymbol{R}}|\Phi\rangle \tag{6}$$

is introduced to the above equation, where $\boldsymbol{A}(t)$ represents the vector potential given by $\boldsymbol{F}(t) = -\frac{d\boldsymbol{A}(t)}{dt}$. This brings Eq. (5) into the following equation for $\Phi_{b\boldsymbol{R}} \equiv \langle b\boldsymbol{R}|\Phi\rangle$ as

$$\sum_{b'\boldsymbol{R}'} e^{-i\boldsymbol{A}(t)\cdot\boldsymbol{R}}\mathcal{H}_{b\boldsymbol{R},b'\boldsymbol{R}'}(t)e^{i\boldsymbol{A}(t)\cdot\boldsymbol{R}'}\Phi_{b'\boldsymbol{R}'} = i\frac{\partial}{\partial t}\Phi_{b\boldsymbol{R}}, \tag{7}$$

where

$$\mathcal{H}_{b\boldsymbol{R},b'\boldsymbol{R}'}(t) = \langle b\boldsymbol{R}|\hat{\mathcal{H}}(t)|b'\boldsymbol{R}'\rangle = H^0_{b\boldsymbol{R},b'\boldsymbol{R}'} + \boldsymbol{F}(t)\cdot\boldsymbol{X}_{bb'}\bar{\delta}_{bb'}\delta_{\boldsymbol{R}\boldsymbol{R}}, \tag{8}$$

in terms of the Hamiltonian $\hat{\mathcal{H}}(t) = \hat{H}^0 + \boldsymbol{F}(t)\cdot\boldsymbol{X}$.

Next, Eq. (7) is rewritten in the Bloch representation, where the Wannier state $|b\boldsymbol{R}\rangle$ is represented in terms of the Bloch state $|b\boldsymbol{k}\rangle$ as

$$|b\boldsymbol{R}\rangle = \frac{1}{\sqrt{N}}\sum_{\boldsymbol{k}} e^{-i\boldsymbol{k}\cdot\boldsymbol{R}}|b\boldsymbol{k}\rangle, \tag{9}$$

with $\boldsymbol{k}$ as the Bloch momentum and N the number of the sites of quantum well. In light of the fact that Eq. (7) is expressed as

$$\sum_{b_1\boldsymbol{R}_1 b_2\boldsymbol{R}_2 b'\boldsymbol{k}'} \langle b\boldsymbol{k}|b_1\boldsymbol{R}_1\rangle\langle b_1\boldsymbol{R}_1|e^{-i\boldsymbol{A}(t)\cdot\boldsymbol{R}_1}\hat{\mathcal{H}}(t)e^{i\boldsymbol{A}(t)\cdot\boldsymbol{R}_2}|b_2\boldsymbol{R}_2\rangle\langle b_2\boldsymbol{R}_2|b'\boldsymbol{k}'\rangle\langle b'\boldsymbol{k}'|\Phi\rangle = i\frac{\partial}{\partial t}\langle b\boldsymbol{k}|\Phi\rangle, \quad (10)$$

one obtains the following equation as

$$\sum_{b'\boldsymbol{k}'}\langle b\boldsymbol{K}(t)|\hat{\mathcal{H}}(t)|b'\boldsymbol{K}'(t)\rangle\tilde{\Phi}_{b'\boldsymbol{k}'} = i\frac{\partial}{\partial t}\tilde{\Phi}_{b\boldsymbol{k}}, \quad (11)$$

with $\boldsymbol{K}(t) \equiv \boldsymbol{k} + \boldsymbol{A}(t), \boldsymbol{K}'(t) \equiv \boldsymbol{k}' + \boldsymbol{A}(t)$ and $\tilde{\Phi}_{b\boldsymbol{k}} \equiv \langle b\boldsymbol{k}|\Phi\rangle$, where in view of Eq. (9), the relations of $\sum_{b_1\boldsymbol{R}_1}\langle b\boldsymbol{k}|b_1\boldsymbol{R}_1\rangle\langle b_1\boldsymbol{R}_1|e^{-i\boldsymbol{A}(t)\cdot\boldsymbol{R}_1} = \langle b\boldsymbol{K}(t)|$ and $\sum_{b_2\boldsymbol{R}_2} e^{i\boldsymbol{A}(t)\cdot\boldsymbol{R}_2}|b_2\boldsymbol{R}_2\rangle\langle b_2\boldsymbol{R}_2|b'\boldsymbol{k}'\rangle = |b'\boldsymbol{K}'(t)\rangle$ are employed. Here, the Hamiltonian matrix element of Eq. (11) is cast into

$$\langle b\boldsymbol{K}(t)|\hat{\mathcal{H}}(t)|b'\boldsymbol{K}'(t)\rangle = \langle b\boldsymbol{K}(t)|\hat{H}^0|b'\boldsymbol{K}'(t)\rangle\delta_{\boldsymbol{k}\boldsymbol{k}'} + \boldsymbol{F}(t)\cdot\boldsymbol{X}_{bb'}\bar{\delta}_{bb'}\delta_{\boldsymbol{k}\boldsymbol{k}'}. \quad (12)$$

Employing the BHZ model for the material Hamiltonian $\hat{H}^0$, Eq. (12) becomes the BHZ Hamiltonian modified by the application of the laser-electron interaction, that is,

$$H(\boldsymbol{k},t) = \mathcal{H}_{\mathrm{BHZ}}(\boldsymbol{K}(t)) + v(t), \quad (13)$$

where a matrix representation is employed with $\mathcal{H}_{\mathrm{BHZ}}(\boldsymbol{K}(t))$ as the BHZ Hamiltonian for the Bloch momentum $\boldsymbol{K}(t)$, and $v(t) \equiv \boldsymbol{F}(t)\cdot\boldsymbol{X}_{sp}$.

Finally, a remark is made on the scheme of calculating edge states supported by bulk bands, when an electron is confined in one direction of the two-dimensional plane of the quantum-well and the other direction remains periodic; such a confined system is equivalent to a semiconductor quantum wire, as seen in Supplementary Figure 1. The Hamiltonian suitable for such a system is obtained by transforming Eq. (7) into a Hamiltonian in terms of a mixed representation using the Wannier representation in one direction and the Bloch representation in the other direction. The resulting wavefunction is represented as $\langle bk_{x(y)}R_{y(x)}|\Phi\rangle$ with $k_{x(y)}$ and $R_{y(x)}$ the $x(y)$- and $y(x)$-components of $\boldsymbol{k}$ and $\boldsymbol{R}$, respectively. The edge states and the associated band structure with respect to $k_{x(y)}$ are calculated under a boundary condition A(B).

5 Supplementary Note 2: Fourier-Floquet matrix elements

The Fourier-Floquet expansion is employed for evaluating the eigenvalue equation given in Eq. (4) of the text. Here, the Floquet Hamiltonian $L(\boldsymbol{k}, t)$ is of the form

$$L(\boldsymbol{k}, t) = \sum_{i=3}^{6} D_i(\boldsymbol{k}, t)\Gamma_i - iI\frac{\partial}{\partial t}, \tag{14}$$

where $D_i(\boldsymbol{k}, t) = d_i(\boldsymbol{K}(t))$ for $i = 3 \sim 5$, and $D_6(\boldsymbol{k}, t) = \Omega_R \cos\omega t$. Explicit expressions of $d_i(\boldsymbol{k})$ are shown in Eq. (2) of the text, and $\boldsymbol{K}(t) = (k_x - \frac{F_x}{\omega}\sin\omega t, k_y)$.

Below, we evaluate the following Floquet matrix (Eq. (5) of the text) as:

$$\tilde{L}_{nn'}(\boldsymbol{k}, \omega) = \frac{1}{T}\int_0^T dt\, e^{-i\Delta n\omega t} L(\boldsymbol{k}, t) = n\omega I\delta_{nn'} + \sum_{i=3}^{6} \tilde{D}_{i,nn'}(\boldsymbol{k}, \omega)\Gamma_i, \tag{15}$$

where

$$\tilde{D}_{i,nn'}(\boldsymbol{k}, \omega) = \frac{1}{T}\int_0^T dt\, e^{-i\Delta n\omega t} D_i(\boldsymbol{k}, t) \tag{16}$$

with $\Delta n = n - n'$. This is expressed in terms of the Nth-order Bessel function of the first kind

$$J_N(z) = \frac{1}{2\pi}\int_0^{2\pi} d\theta\, e^{-iN\theta} e^{iz\sin\theta} \tag{17}$$

with $z = F_x a/\omega$ (a: a lattice constant). Therefore, we obtain

$$\tilde{D}_{3,nn'}(\boldsymbol{k}, \omega) = \begin{cases} 2t_{sp}\sin k_y a & \text{for } \Delta n = 0 \\ 0 & \text{for } \Delta n \neq 0 \end{cases}, \tag{18}$$

$$\tilde{D}_{4,nn'}(\boldsymbol{k}, \omega) = \begin{cases} \frac{1}{2}(\epsilon_s - \epsilon_p) - (t_{ss} + t_{pp})(J_0(z)\cos k_x a + \cos k_y a) & \text{for } \Delta n = 0 \\ -(t_{ss} + t_{pp})J_{\Delta n}(z)\cos k_x a & \text{for } \Delta n = \pm 2, \pm 4, \cdots \\ i(t_{ss} + t_{pp})J_{\Delta n}(z)\sin k_x a & \text{for } \Delta n = \pm 1, \pm 3, \cdots \end{cases}, \tag{19}$$

$$\tilde{D}_{5,nn'}(\boldsymbol{k}, \omega) = \begin{cases} 2t_{sp}J_{\Delta n}(z)\sin k_x a & \text{for } \Delta n = 0, \pm 2, \pm 4, \cdots \\ 2it_{sp}J_{\Delta n}(z)\cos k_x a & \text{for } \Delta n = \pm 1, \pm 3, \cdots \end{cases}, \tag{20}$$

and

$$\tilde{D}_{6,nn'}(\boldsymbol{k}, \omega) = \begin{cases} \frac{1}{2}\Omega_R & \text{for } \Delta n = \pm 1 \\ 0 & \text{for } \Delta n \neq \pm 1 \end{cases}. \tag{21}$$

Let a matrix element with component $(b(n), b'(n'))$ of $\tilde{L}_{nn'}(\boldsymbol{k}, \omega)$ be represented as $\tilde{L}_{b(n)b'(n')}(\boldsymbol{k}, \omega)$. Given Eq. (15) and the above expressions of $\tilde{D}_{i,nn'}(\boldsymbol{k}, \omega)$ ($i = 3 \sim 6$), it is readily seen that $\tilde{L}_{b(n)b'(n')}(\boldsymbol{k}, \omega)$ vanishes if either Δn is an odd number and $b = b'$, or Δn is an even number and $b \neq b'$.

6 Supplementary Note 3: Additional condition of quasienergy band crossing

The origin of quasienergy band crossings is examined. It is expected that because of the conservation of both T- and pseudo-I-symmetries, the band crossings occur at the high-symmetry points [1]. Below, we show that an additional condition is required that is pertinent to the difference of the photon numbers between two bands participating to the crossing.

6.1 A σ_z-conserving interaction

Since different spin states are decoupled, namely, $[L(\boldsymbol{k},t), I_2 \otimes \sigma_z] = 0$, just an upper-spin diagonal block-matrix of $\tilde{L}_{nn'}(\boldsymbol{k},\omega)$, denoted as $\tilde{L}^{\uparrow\uparrow}_{nn'}(\boldsymbol{k},\omega)$, suffices for this purpose. Equation (5) of the text is solved to seek an eigenvalues $E_\alpha(\boldsymbol{k}^j)$ at a high-symmetry point $j = \Gamma, X_2$, and X_1. Here we consider how one eigenvalue $E_{b(n)}(\boldsymbol{k}^j)$ of state $b(n)$ is affected by another state $b'(n')$. The state $b(n)$ is coupled with $b'(n')$ through a set of sequential interactions composed of products of matrix element $V^{(n_i-n_j)}_{b_i(n_i)b_j(n_j)} \equiv \tilde{L}^{\uparrow\uparrow}_{b_i(n_i)b_j(n_j)}(\boldsymbol{k}^j,\omega)$. Let the mth-order sequential interaction for couplings between the two states $b(n)$ and $b'(n')$ be represented as a matrix element $U^{(m)}_{b(n)b'(n')}$. That is,

$$U^{(m)}_{b(n)b'(n')} = \sum_{b_2(n_2),\cdots,b_m(n_m)} \prod_{i=1}^{m} V^{(n_i-n_{i+1})}_{b_i(n_i)b_{i+1}(n_{i+1})}, \tag{22}$$

where $b_1(n_1) \equiv b(n)$, $b_{m+1}(n_{m+1}) \equiv b'(n')$, and a net change of photon numbers carried by $U^{(m)}_{b(n)b'(n')}$ is identical with $\Delta n \equiv n - n'$: $\sum_{i=1}^{m}(n_i - n_{i+1}) = \Delta n$. As is concluded in Supplementary Note 1, $V^{(n_i-n_j)}_{b_i(n_i)b_j(n_j)} = 0$ if either $(n_i - n_j)$ is an odd number and $b_i = b_j$, or $(n_i - n_j)$ is an even number and $b_i \neq b_j$; otherwise $V^{(n_i-n_j)}_{b_i(n_i)b_j(n_j)} \neq 0$.

Below, it is inductively proved that $U^{(m)}_{p(n)s(n-\Delta n)} = U^{(m)}_{s(n)p(n-\Delta n)} = 0$ if Δn is even, and $U^{(m)}_{b(n)b(n-\Delta n)} = 0$ if Δn is odd; otherwise $U^{(m)}_{b(n)b'(n')} \neq 0$. To begin with, it is verified that these relations hold for $m = 1, 2$, based on the above-mentioned properties of $V^{(n_i-n_j)}_{b_i(n_i)b_j(n_j)}$. First, since $U^{(1)}_{p(n)s(n-\Delta n)}$ is given by

$$U^{(1)}_{p(n)s(n-\Delta n)} = V^{(\Delta n)}_{p(n)s(n-\Delta n)}, \tag{23}$$

evidently it vanishes for Δn even, and so does $U^{(1)}_{s(n)p(n-\Delta n)}$. Further, since

$$U^{(1)}_{b(n)b(n-\Delta n)} = V^{(\Delta n)}_{b(n)b(n-\Delta n)}, \tag{24}$$

this also vanishes for Δn odd. Second, bearing in mind that $U^{(2)}_{p(n)s(n-\Delta n)}$ is expanded as

$$U^{(2)}_{p(n)s(n-\Delta n)} = \sum_{b_2(n_2)} \left[U^{(1)}_{p(n)b_2(n_2)} U^{(1)}_{b_2(n_2)s(n-\Delta n)} \right], \tag{25}$$

if Δn is even, either $U^{(1)}_{p(1)b_2(n_2)}$ or $U^{(1)}_{b_2(n_2)s(n-\Delta n)}$ vanishes for any set of indices of b_2 and n_2. Thus $U^{(2)}_{p(n)s(n-\Delta n)}$ vanishes, and so does $U^{(2)}_{s(n)p(n-\Delta n)}$. Similarly,

$$U^{(2)}_{b(n)b(n-\Delta n)} = \sum_{b_2(n_2)} \left[U^{(1)}_{b(n)b_2(n_2)} U^{(1)}_{b_2(n_2)b(n-\Delta n)} \right], \tag{26}$$

vanishes, if Δn is odd.

Next, suppose for $k \geq 3$ that $U^{(k)}_{p(n)s(n-\Delta n)} = U^{(k)}_{s(n)p(n-\Delta n)} = 0$ for Δn even, and $U^{(k)}_{b(n)b(n-\Delta n)} = 0$ for Δn odd. Now, $U^{(k+1)}_{p(n)s(n-\Delta n)}$ is considered, which is read as

$$U^{(k+1)}_{p(n)s(n-\Delta n)} = \sum_{l=1}^{k} \sum_{b_2(n_2)} U^{(l)}_{p(n)b_2(n_2)} U^{(k+1-l)}_{b_2(n_2)s(n-\Delta n)}. \tag{27}$$

Here, if Δn is even, either $U^{(l)}_{p(n)b_2(n_2)}$ or $U^{(k+1-l)}_{b_2(n_2)s(n-\Delta n)}$ vanishes for any set of indices of b_2 and n_2, because $l, k+1-l \leq k$ for $l = 1 \sim k$. Thus, $U^{(k+1)}_{p(n)s(n-\Delta n)} = 0$ for Δn even, and so does $U^{(k+1)}_{s(n)p(n-\Delta n)}$. Similarly, it is obtained that $U^{(k+1)}_{b(n)b(n-\Delta n)} = 0$ for Δn odd. Therefore, it has been proved that for any order of m, $U^{(m)}_{p(n)s(n-\Delta n)} = 0$ and $U^{(m)}_{s(n)p(n-\Delta n)} = 0$ when Δn is even, and $U^{(m)}_{b(n)b(n-\Delta n)} = 0$ when Δn is odd; otherwise $U^{(m)}_{b(n)b'(n-\Delta n)} \neq 0$.

Accordingly, due to $U^{(m)}_{p(1)s(-1)} = 0$, $E_{p(1)}(\boldsymbol{k}^j)$ and $E_{s(-1)}(\boldsymbol{k}^j)$ are given by a series expansion just in terms of self-energies $U^{(m)}_{p(1)p(1)}$ and $U^{(m)}_{s(-1)s(-1)}$, respectively. Thus, it is found that the crossings between these two bands occur at F^j_x ensuring a single algebraic equation $E_{p(1)}(\boldsymbol{k}^j) = E_{s(-1)}(\boldsymbol{k}^j)$. In view of the spin degree of freedom, $p(1)$ and $s(-1)$ are four-fold degenerate at $\boldsymbol{k}^j$'s. This result holds correct for any interband coupling, in general, between $p(n)$ and $s(n')$ if $(n - n')$ is even, while for odd numbers of $(n - n')$, s- and p-bands are gapped out; especially, $p(1)$ and $s(0)$ are anticrossed due to the leading interaction $V^{(1)}_{p(1)s(0)}$ of the order of Ω_R.

6.2 A σ_z-non-conserving interaction

Next, we consider the effect of a σ_z-non-conserving interaction on the obtained results, which is given by

$$v_z(\boldsymbol{k}, t) = \sum_{i=1,2} D_i(\boldsymbol{k}, t)\Gamma_i, \tag{28}$$

where $D_i(\boldsymbol{k}, t) = d_i(\boldsymbol{K}(t))$, and $d_i(\boldsymbol{k})$'s are odd functions with respect to $\boldsymbol{k}$ to keep $v_z(\boldsymbol{k}, t)$ both T- and pseudo-I-symmetric. Based on the two-component model, when Δn is even, it is evident that a gap remains closed because of $\tilde{D}_{i,n_0 n'_0}(\boldsymbol{k}^j, \omega) = 0$ with $i = 1, 2$. Similarly to the above case of the σ_z-conserving interactions, this still hold in general, as is verified in Supplementary Note 2.

Next, we examine the effect of a σ_z-non-conserving interaction on the obtained results, the interaction of which is given by Eq. (6) of the text as

$$v_z(\boldsymbol{k}, t) = \sum_{j=1,2} D_j(\boldsymbol{k}, t)\Gamma_j \tag{29}$$

with $D_j(\boldsymbol{k}, t) = d_j(\boldsymbol{K}(t))$. Here, $d_1(\boldsymbol{k})$ and $d_2(\boldsymbol{k})$ are odd function with respect to $\boldsymbol{k}$ to keep $v_z(\boldsymbol{k}, t)$ both T- and pseudo-I-symmetric. This coupling between the same spin states of $p(1)$ and $s(-1)$ is conducted by series of interactions composed of a spin inversion due to

$$\tilde{v}_{z,nn'}(\boldsymbol{k}, \omega) = \frac{1}{T} \int_0^T dt\, e^{-i(n-n')\omega t} v_z(\boldsymbol{k}, t). \tag{30}$$

and the σ_z-conserving coupling $U^{(m)}_{b(n)b'(n')}$. It is evident that $\tilde{v}_{z,nn'}(\boldsymbol{k}^j, \omega) \neq 0$ if $(n - n')$ is odd, otherwise it vanishes, and thus $\tilde{v}_{z,nn'}(\boldsymbol{k}^j, \omega)$ is subject to the property similar to $V^{(n-n')}_{b(n)b'(n')}$ with $b \neq b'$. Therefore, the coupling of concern between $p(1)$ and $s(-1)$ is also provided by the form of the sequential interaction modified from $U^{(m)}_{p(1)s(-1)}$ just in that even number of times spin-inversion interactions are incorporated with $U^{(m)}_{b(n)b'(n')}$. This leads us to the conclusion that these two bands remain gapless.

6.3 Summary

For the realization of band crossings at $\boldsymbol{k}^j$'s in the concerned Floqeut system, we require the additional condition that the difference of photon numbers between participating bands be an even number with the condition of the conservation of both T- and pseudo-I-symmetries

[1] Murakami, S. *et al.* Tuning phase transition between quantum spin Hall and ordinary insulating phases. *Phys. Rev. B* **76** 205304 (6 pages) (2007).

7 Supplementary Note 4: The degree of localization of the edge states in the vicinity of $F_x^{X_2}$

Here, we examine the variance of the degree of localization of the edge states in the vicinity of the $\bar{X}_2$ point ($k_x = 0$), as F_x changes around $F_x^{X_2}$; the results shown right below are also applicable to the edge states emerging at F_x in the vicinity of $F_x^{X_1}$. To do this, the electron density is defined as

$$\rho_{k_x}(R_y) = \frac{1}{T}\int_0^T dt \sum_b |\langle bk_xR_y|\Psi_{k_x\alpha}(t)\rangle|^2 , \tag{31}$$

where $\Psi_{k_x\alpha}(t)$ represents the αth eigenvector of Eq. (4) of the text under the boundary condition A, and $|bk_xR_y\rangle$ is a basis in the mixed representation, as mentioned in Supplementary Note 1. It is obvious that $\sum_{R_y}\rho_{k_x}(R_y) = 1$. Here, α is set an edge state emerging at each F_x given below.

The calculated results of $\rho_{k_x}(R_y)$ are shown in Fig. 4 of the present Note alongside the associated $\mathcal{E}_\alpha(k_x)$. As shown in the panel (a), the topological edge state (shown in the panel (d)) remains localized in the edge region irrespective of the change of k_x. As shown in the panel (b), the Dirac-Tamm state (shown in the panel (e)) has the same pattern of change as the above topological edge state, because both of the edge states originate from the band inversion at the Γ point; see Figs. 5d and 5e of the text. However, there is an exceptional case that the Dirac-Tamm state becomes delocalized at $k_x/\pi = 0.025$ just close to the $\bar{X}_2$ point. Such a property is caused by an interaction of this state with the Floquet DSM continuum phase at the X_2 point. To be more specific, this interaction is attributed to an almost energetically degenerate coupling between the Γ and X_2 points, because k_y is no longer a good quantum number in this 1D confined system, and states with different k_y's are likely coupled.

As regards the Tamm state (shown in the panel (f)), it is shown in the panel (c) that this is delocalized in the whole R_y-region and disappears in a local region of k_x where the anticrossing around the X_2 point occurs ($k_x/\pi = 0.025$ and 0.05), though this is still localized in a region where $|k_x|$ is greater ($k_x/\pi = 0.1$). In fact, due to this anticrossing, the parity hybridization is strongly reduced, leading to the absence of an edge state. It is evident from Fig. 5f of the text that the anticrossing formation around the X_2-point gives rise to the sign inversion of $\tilde{\mathcal{D}}(\boldsymbol{k})$ — from $\tilde{\mathcal{D}}(\boldsymbol{k}) > 0$ seen in Figs. 5d and 5e of the text — and the zero contour vanishes in the whole k_y region with k_x assigned around the $\bar{X}_2$ point. Thus, an edge state does not exist in this local k_x region.

To summarize, all edge states (a topological edge state, a Dirac-Tamm state, and a Tamm state) extending over k_x regions provided by the projection of the zero contours are localized in the y direction of the quantum well, and properties pertinent to the localization seem unaltered with the change of F_x around $F_x^{X_2}$, whether there is a Dirac node or not in an edge state. However, it is noted that the Dirac-Tamm state is made delocalized in the close vicinity of $\bar{X}_2$ due to the interaction with the associated Floquet DSM phase.

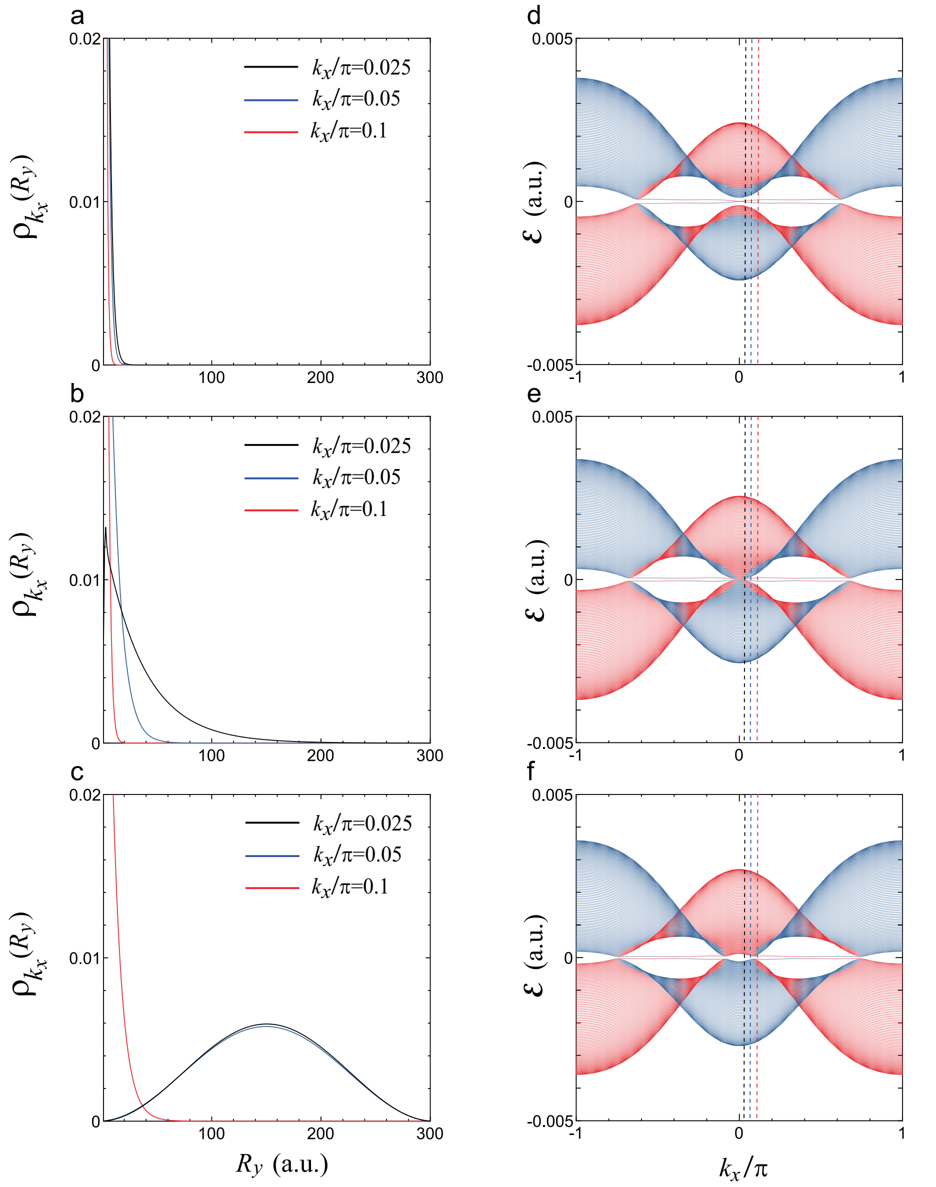

Figure 4: **The degree of localization of the edge states in the vicinity of $F_x^{X_2}$. (a)** The electron density $\rho_{k_x}(R_y)$ of edge states close to $\mathcal{E}(k_x) = E_F$ as a function of R_y with k_x/π equal to 0.025 (black solid line), 0.05 (blue solid line), and 0.1 (red solid line) at $F_x = 2.14 \times 10^{-4}$ a.u. (1.10 MV/cm) corresponding to Fig. 5a in the text ($F_x^{X_2} < F_x < F_x^{\Gamma}$). **(b)** The same as the panel (a) but at $F_x = 2.04 \times 10^{-4}$ a.u. (1.05 MV/cm) corresponding to Fig. 5b in the text ($F_x = F_x^{X_2}$). **(c)** The same as the panel (a) but at $F_x = 1.94 \times 10^{-4}$ a.u. (997 kV/cm) corresponding to Fig. 5c in the text ($F_x^{X_1} < F_x < F_x^{X_2}$). **(d)** Quasienergy bands $\mathcal{E}(k_x)$ as functions of k_x for the reference of the panel (a), where the positions of k_x/π equal to 0.025 (black dotted line), 0.05 (blue dotted line), and 0.1 (red dotted line) are also depicted. **(e)** The same as the panel (d) but for the reference of the panel (b). **(f)** The same as the panel (d) but for the reference of the panel (c).
